# One round of azithromycin MDA adequate to interrupt transmission in districts with prevalence of trachomatous inflammation—follicular of 5.0-9.9%: Evidence from Malawi

**DOI:** 10.1371/journal.pntd.0006543

**Published:** 2018-06-13

**Authors:** Khumbo Kalua, Alvin Chisambi, David Chinyanya, Michael Masika, Ana Bakhtiari, Rebecca Willis, Paul M. Emerson, Anthony W. Solomon, Robin L. Bailey

**Affiliations:** 1 Department of Ophthalmology, University of Malawi, College of Medicine, Blantyre, Malawi; 2 Blantyre Institute for Community Ophthalmology, Lions Sight First Eye Hospital, Blantyre, Malawi; 3 Ministry of Health, Lilongwe, Malawi; 4 International Trachoma Initiative, Task Force for Global Health, Decatur, Georgia, United States of America; 5 Department of Control of Neglected Tropical Diseases, World Health Organization, Geneva, Switzerland; 6 Clinical Research Department, London School of Hygiene & Tropical Medicine, London, United Kingdom; Harvard Medical School, UNITED STATES

## Abstract

**Background:**

As highly trachoma-endemic countries approach elimination, some districts will have prevalences of trachomatous inflammation–follicular in 1–9-year-olds (TF_1-9_) of 5.0–9.9%. The World Health Organization (WHO) previously recommended that in such districts, TF prevalence be assessed in each sub-district (groupings of at least three villages), with three rounds of azithromycin treatment offered to any sub-district in which TF≥10%. Given the large number of endemic districts worldwide and the human and financial resources required to conduct surveys, this recommendation may not be practical. In a group of 8 Malawi districts with baseline TF prevalences of 5.0–9.9%, the Malawi Ministry of Health administered one round of azithromycin mass treatment, to the whole of each district, achieving mean coverage of ~80%. Here, we report impact surveys conducted after that treatment.

**Methods:**

We undertook population-based trachoma surveys in 18 evaluation units of the 8 treated districts, at least 6 months after the MDA. The standardized training package and survey methodologies of Tropical Data, which conform to WHO recommendations, were used.

**Results:**

Each of the 18 evaluation units had a TF_1-9_ prevalence <5.0%.

**Conclusion:**

The study demonstrates that in Malawi districts with TF of 5.0–9.9%, one round of azithromycin MDA with ~80% coverage associates with a reduction in TF prevalence to <5%. Further evidence for this approach should be collected elsewhere.

## Author summary

Until now, in trachoma elimination programmes, the WHO recommendation for district-wide annual rounds of antibiotic mass drug administration was only applicable to districts with a trachomatous inflammation—follicular (TF_1-9_) prevalence of 10% or more. Districts with a TF_1-9_ prevalence of <5% were considered not to require intervention with antibiotics for trachoma. For districts with a prevalence of 5.0–9.9%, programmes were encouraged to determine the TF_1-9_ prevalence at sub-district or “community” level. With the recent rapid scale-up in trachoma mapping, there are now a large number of districts known to have TF_1-9_ prevalence of 5.0–9.9%, so this recommendation is likely to pose an implementation challenge to health ministries and their partners. In this study, we have demonstrated that in districts with TF_1-9_ 5.0–9.9%, a single round of mass drug administration with high coverage to the whole district can be followed by an impact survey TF_1-9_ prevalences of <5%. This approach is likely to reduce the commodity need (one round of MDA to 100% of the population compared to 3 or more rounds to an average 50% of the population), reduce the number of surveys required, and ultimately accelerate the speed of progress to elimination. We recommend that this finding be further explored elsewhere to determine its generalizability, in order to justify consideration of global policy change.

## Introduction

Trachoma is thought to be a significant public health issue in at least 42 countries [1]. It is caused by repeated ocular re-infection [2] with serotypes A, B, Ba and C of the bacterium *Chlamydia trachomatis* [3,4]. Resolution of episodes of active (inflammatory) trachoma, which are associated with *C*. *trachomatis* infection, is accompanied by sub-epithelial scarring of the eyelid. Scarring may eventually lead to trichiasis; trichiasis can irreversibly impair vision. Many endemic countries have made considerable progress towards elimination of trachoma as a public health problem, [5,6] thanks to the robust commitment of governments and their partners. The target for global elimination is the end of the year 2020[7].

Active trachoma is controlled through implementation of the A (antibiotics), F (facial cleanliness) and E (environmental improvement) components of the WHO-endorsed “SAFE strategy” [8]; the S (surgery) component serves to manage individuals who have developed trichiasis. From a trachoma elimination perspective, the need for implementation of the A, F and E components is guided by the district-level prevalence of the active trachoma sign trachomatous inflammation—follicular (TF) [9] in 1–9-year-olds, where “districts” are defined as populations of 100,000–250,000 people, [10] and TF prevalence in a district is determined through a population-based survey. [11] WHO recommends that where the district-level TF prevalence is ≥10%, A, F and E, including annual district-wide mass drug administration (MDA) of antibiotics, should be implemented for at least three years before re-survey[11, 12].

The programmatic goal is to reduce the district-level prevalence of TF in 1–9-year-old children to <5% [5]. In previously published [10] advice, WHO suggested that in districts in which the TF prevalence in 1–9-year-olds is estimated to be <10% but greater than 5%—the so-called 5–9% districts-, TF prevalence should be re-estimated at sub-district-level, with sub-districts defined as “geographic or other grouping of at least three villages [permitting] finer stratification of a district into sub-units that might be expected to have greater or lesser prevalence of trachoma”. Following the recent completion of the Global Trachoma Mapping Project (GTMP), [13, 14] it was calculated that there were 1466 districts worldwide in which the TF prevalence in 1-9-year-olds was ≥5%. At some point in the elimination pathway (in a population’s journey from active-trachoma-is-a-public-health-problem to eliminated-active-trachoma-as-a-public-health-problem), many of those 1466 districts would enter the 5–9% grey area. The expense, and time required to divide these districts into sub-districts and re-estimating TF prevalence at finer resolution would be considerable.

In line with the global target, Malawi seeks national elimination of trachoma by the year 2020. Eight Malawi districts had baseline TF prevalences of 5.0–9.9%, obtained from surveys conducted in 2013 using the Global Trachoma Mapping Project (GTMP) methodology. Rather than segmenting and re-surveying these districts, the Malawi Ministry of Health applied to the International Trachoma Initiative for, and received, a donation of one round of azithromycin for MDA across the entirety of each district. MDA was conducted between October and November 2015, to a total target population of 3,632,175. As previously reported elsewhere, [15] antibiotic coverage (Table 1) was determined by the Ministry of Health and partners through both routine service data collection and dedicated post-MDA coverage surveys; the latter estimated the mean district-level azithromycin and tetracycline (TEO) coverage at 78.9% (range 69.5–83.9%). (WHO recommends coverage of at least 80%, [11] which was achieved in 5 of 8 districts.) An opportunity then arose to examine the impact of a single round of mass antibiotic treatment within a large trachoma-hypo endemic population. In this paper, we report on the post-MDA impact surveys conducted.

**Table 1:** Trachomatous inflammation—Follicular (TF) prevalence at baseline and at impact survey, selected districts, Malawi, 2013–2016.

Region	District (2016population estimate)	Year of baseline survey	TF prevalence in 1–9 year-olds at baseline (%) [95% CI] [17]	Water coverage at baseline, %	Sanitation coverage at baseline, %	Antibiotic coverage (November 2015, %)[15]	Water coverage at impact survey, %	Sanitation coverage at impact survey, %	TF prevalence in 1–9 year-olds at impact survey, % [95% CI]
Central	Dowa (592,384)	2013	8.3 [5.3–12.4]	70.0	5.6	81.5	82.10	5.56	1.5 [1.1–2.1]
	Lilongwe West (777,221)	2013	9.9 [6.9–13.9]	87.6	4.8	83.9	57.39	8.57	1.8 [1.3–2.3]
	Ntcheu (534,168)	2013	6.0 [3.7–8.1]	80.8	3.5	69.5	82.09	4.74	1 [0.7–1.5]
	Ntchisi (252,297)	2013	7.8 [5.5–10.5]	82.7	9.2	74.5	90.47	4.97	0.2 [0–0.6]
Southern	Machinga (550,529)	2013	7.2 [4.0–11.6]	83.8	3.1	72.1	79.75	4.83	1.9 [1.4–2.4]
	Mwanza (105,364)	2013	7.8 [6.6–9.2]^45^	79.8	2.4	83.2	88.77	6.66	2.1 [1.3–3.2]
	Neno (119,608)	2013	6.8 [4.2–9.6]	81.5	5.9	81.8	86.51	4.59	0.4 [0.1–1.1]
	Zomba Rural (340,567)	2013	5.3 [2.9–9.1]	90.1	10.1	83.6	91.85	8.21	0.9 [0.6–1.4]

## Methodology

In eight Malawi districts in which the baseline TF prevalence had been 5.0–9.9%, and in which a single round of antibiotic MDA had then been undertaken, we conducted a cross sectional study: a series of impact surveys to re-estimate the prevalence of TF. Fieldwork was carried out between May and August 2016, with each survey occurring at least 6 months after MDA.

A possible point of semantic confusion arises in describing these surveys, because six of eight Malawi districts had populations larger than the model “district” of 100,000–250,000 residents recommended[10] by WHO as the population unit for conducting impact surveys. We split the six Malawi districts with populations larger than 250,000 into smaller population units (Table 2) for the purposes of conducting the surveys. Although these population units would be considered “sub-districts” in Malawi, they are not sub-districts in the sense defined by the (2010) Third Global Scientific Meeting on Trachoma, which was the basis for recent WHO advice [10] on this topic, because creating WHO-style sub-districts would have required division into “geographic or other grouping of at least three villages” encompassing populations of <100,000 people. [10] To be clear, using WHO definitions, the surveys reported here are “district-level” rather than “sub-district-level” surveys.

**Table 2:** Evaluation unit population sizes, number of 1–9-year-old children examined, and trachomatous inflammation—Follicular (TF) prevalence in 1–9-year-olds, impact surveys, Malawi, May–August 2016.

Original district (at baseline)	Evaluation unit (2016)	2016 population estimate	Number of 1–9-year-olds examined	TF prevalence in 1–9-year-olds (%) [95% CI]
Dowa	Dowa Mponela	188,112	886	1.1 (0.2–2.1)
	Dowa Madisi	188,459	941	2.4 (1.3–3.5)
	Dowa Central	215,813	922	1.2 (0.2–2.4)
Lilongwe	Lilongwe Kalolo	259,000	879	1.1 (0.3–2.1)
	Lilongwe Kasiya	259,100	812	2.1 (1.1–3.5)
	Lilongwe Kabudula	259,121	870	1.6 (0.7–2.2)
Ntcheu	Ntcheu Lizulu	253,607	897	0.6 (0.1–1.2)
	Ntcheu Tsangano	116,399	984	0.6 (0.3–1.0)
	Ntcheu Bwanje	251,095	986	1.2 (0.5–2.0)
Ntchisi	Ntchisi DHO	141,206	855	0.2 (0.0–0.5)
	Ntchisi Malomo	130,000	848	0.1 (0.0–0.3)
Machinga	Machinga DHO	186,153	981	1.5 (0.7–2.5)
	Machinga Ntaja	243,140	1,130	0.9 (0.4–1.5)
	Machinga Mpiri	315,845	1,072	2.9 (1.6–4.4)
	Mwanza	106,493	956	2.5 (1.2–4.2)
	Neno	121,070	916	0.3 (0.0–0.8)
Zomba	Zomba Mayaka Rural	214,042	989	0.8 (0.2–2.0)
	Zomba Rural Likangala	183,520	1,174	0.8 (0.2–1.5)

To avoid possible confusion between the term used for the Malawi administrative division and the WHO-recommended population unit, in this paper, we will henceforth refer to each impact survey area as an “evaluation unit” (EU). A further point to note is that although 18 EUs were created for conducting impact surveys, MDA had been undertaken and monitored by the Ministry of Health and its partners at the level of the Malawi district (Table 2): we have neither EU-level baseline TF prevalences nor EU-level MDA coverage figures because those activities were not powered at EU level.

### Sample size, and selection of clusters, households and individuals

Each impact survey was designed to obtain the EU-level prevalence estimate for TF in children aged 1–9 years. The sample size for each EU was calculated to estimate, with 95% confidence, an expected TF prevalence of 4% with absolute precision of 2%.10 In 200 population-based trachoma prevalence surveys conducted with the support of the GTMP in which the prevalence of TF in 1–9-year-olds turned out to be <5%, the 75th centile of individual-survey design effects (from smallest to biggest) was 2.71 (Macleod et al, manuscript in preparation). Using this design effect, and the single population proportion for precision formula,16 1000 children aged 1–9 years should be included; inflating by 1.2 to account for non-response, the required sample size to frame in a trachoma impact survey is 1200 children aged 1–9 years. To achieve this sample size in the Malawi context, 30 households from each of 24 villages (clusters; mean population 1000–2000 residents) were needed from each EU. In each EU, therefore, a list of all villages was obtained from the District Health Office, and 24 were selected systematically, with probability of selection proportional to population size [16]. To ensure the “probability of selection proportional to Population size”, a list of all the clusters (villages) and their respective population sizes was produced on an excel sheet. A column was created with the cumulative population across the enumeration areas and the total population was divided by the number of clusters (24) required to derive the sampling interval. The first cluster was selected by multiplying the sampling interval with a random number between 0 and 1, the resulting number was traced in the cumulative population column, and the first cluster was chosen as the corresponding village. Consecutive clusters (village) were identified by adding the sampling interval to the previous number.

Finally, in each sampled village, all households were listed and 30 were selected using computer-generated random numbers. All residents of selected households aged 1 year or more were invited to participate.

### Training

The standardized training systems and methodologies of Tropical Data, [18] which conform with WHO guidelines, [19] were used. Training of graders, recorders and supervisors was conducted in Mangochi District, Malawi, by a Tropical Data-certified Master Grader Trainer (KK) and a Tropical Data-certified Recorder Trainer (AC). All grader trainees had previously been GTMP-certified [17, 20]. Grader trainees participated in two days of refresher training, which covered methods for examining subjects for TF, trachomatous inflammation—intense (TI) and trachomatous trichiasis (TT), and the recognition of and referral pathways for other diseases. Recorders were taught how to use the Tropical Data Android-based data collection app. Training consisted of both theoretical classroom lessons and field practice; in the latter, certified graders and recorders practiced together. Fifteen teams were formed, each consisting of one grader and one recorder.

### Field methods

Each cluster was surveyed by one team in one day. Selected clusters were visited a few days in advance of the scheduled survey date by a Health Surveillance Assistant (HSA) from the Ministry of Health, whose role was to brief the village chief and community members and prepare a list of households. When the survey team arrived in the village, that list was used to determine the randomly selected households. Numbered household listing had already been prepared by the HSA prior to teams arriving in the field, and the teams used the printed random numbers selected from the computer to assign on the paper list, corresponding numbers of households chosen, prior to the field work starting. The survey team then moved from one selected house to the next. After obtaining written consent from the household head and informing the study participants, global positioning system and water, sanitation and hygiene (WASH) data were collected at household level, household residents were enumerated, and consenting household residents aged ≥1 year were examined for signs of trachoma using a 2.5× magnifying loupe (Binomag plastic, USA) and sunlight[13]. Individuals found to have active trachoma were offered two 3.5g tubes of 1% tetracycline eye ointment and adults with trichiasis were referred to the district hospital where free trichiasis surgery was available.

#### Quality control

The work of survey teams was overseen by two supervisors, Ministry of Health staff and the master trainer. Supervisors were experienced Ophthalmic Clinical Officers who had been certified as grader trainers [13] and were part of the training team. At the completion of field work for each EU, graders, recorders and supervisors met to discuss logistical challenges faced and suggest solutions.

### Data analyses

Analyses were conducted in R and Microsoft Excel. Our primary outcome measure for the purposes of this study was the TF prevalence in 1–9-year-olds in each EU. Proportions of children with TF in each cluster were adjusted for age in one-year age bands, with data from the most recent census used as a reference. The EU-level TF prevalence was the mean of the adjusted cluster-level proportions. Confidence intervals were determined by bootstrapping, with 10,000 iterations [21]. To permit comparisons between TF prevalence at baseline and at impact survey, Malawi district–level TF prevalences were also generated by producing population-weighted means of the EU-level prevalences within each district.

### Ethics

Ethical permission for trachoma studies was obtained from the Malawi National Ethics committee of the Ministry of Health and the London School of Hygiene & Tropical Medicine (6319 and 8355).

## Results

In the 18 EUs, a total of 432 clusters were selected, as per protocol. In total, from the 12,960 households, 28,095 children aged 1–9 were enrolled, among which 26,158 (93%) were examined. Consent for examination was refused for 120 children; 1,810 children were absent at the time of the survey team’s visit; and 7 children were ill and not examined. The analysis focused only on the children examined.

All EUs had a TF prevalence in 1–9 year-olds of <5.0%, i.e., below the WHO-defined threshold for elimination of active trachoma,^5^ with the lowest having a prevalence of 0% and the highest 2.9%. The upper bound of the 95% confidence interval for each TF prevalence estimate was <5.0% (Table 1).

**Fig 1:**
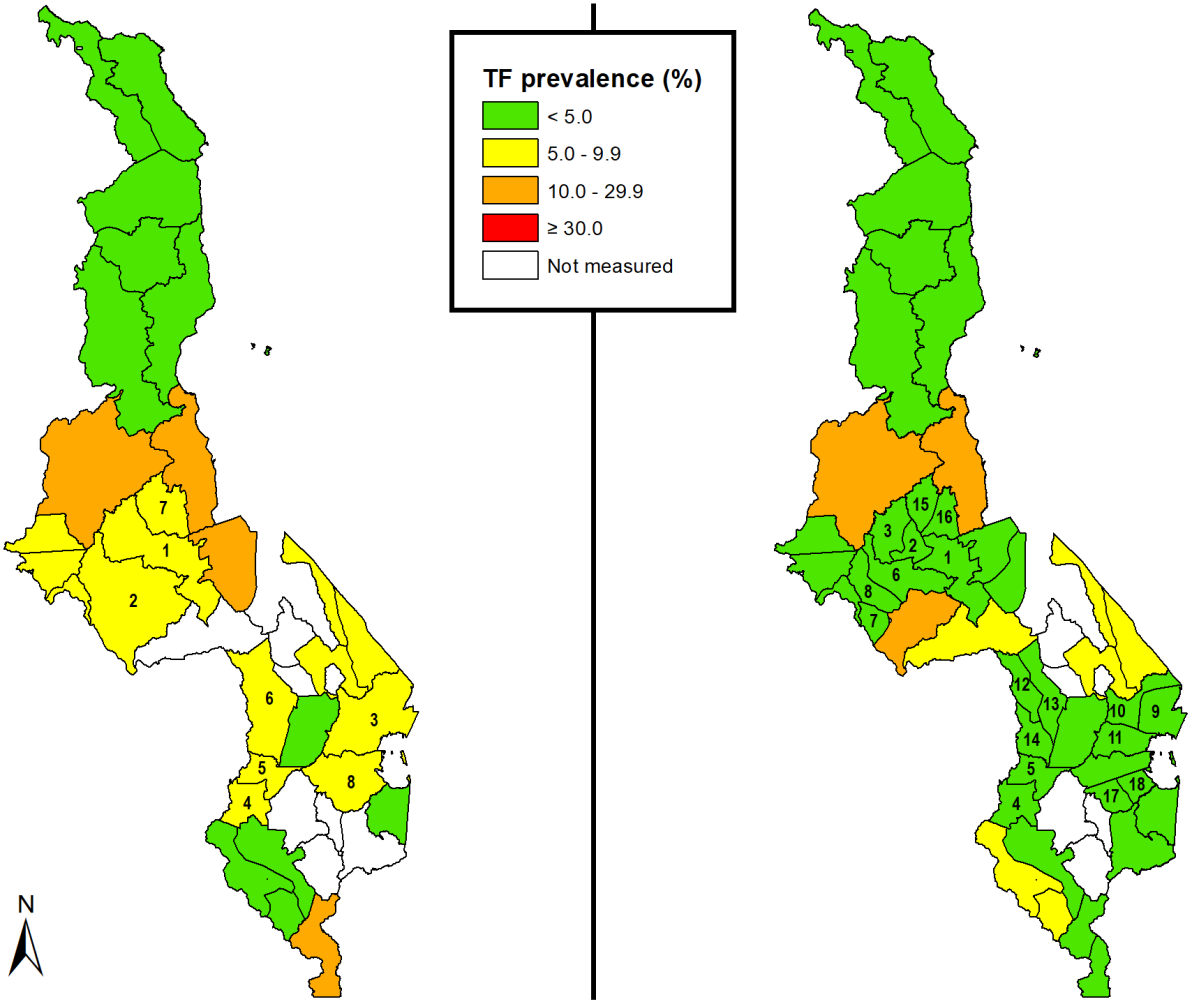
Comparison of the most recent prevalence estimates for trachomatous inflammation—Follicular (TF) in 1–9-year-olds, Malawi, in (a) 2014 and (b) 2016 (after the impact surveys reported here, undertaken following one round of azithromycin MDA).

Fig 1 contrasts TF prevalences at baseline (2012–2014), and after impact surveys (2016) following one round of azithromycin MDA. Its first panel shows district-level baseline TF prevalences as of 2014, with yellow shading indicating the districts in which TF prevalence was 5.0–9.9%. Its second panel depicts the TF prevalence map in 2016, taking into account the current tranche of EU-level impact survey results. The areas in which the TF prevalence is shown as being 5.0–9.9% are those EUs that, at the time of preparation of this manuscript, had (with one exception) already received recently azithromycin MDA but had not yet had impact surveys completed. The areas shown as having TF prevalence estimates ≥10% had all completed three rounds of azithromycin MDA and were awaiting impact surveys.

## Discussion

Having set its sights on eliminating trachoma as a public health problem nationally by 2020, Malawi was challenged by the realisation that a number of its constituent districts had TF prevalences in a range for which WHO guidance suggested higher resolution mapping, rather than immediate public health action which would have added up to five years to the attainment of the elimination target and pushing the likely date of elimination beyond 2020. Fortunately, a considerable body of evidence was available to suggest that azithromycin MDA is both effective in lowering the prevalence of active trachoma [22] and associated with a very low rate of severe adverse events[23–25]. There is also expanding evidence for a wide range of desirable ancillary effects of azithromycin MDA in populations receiving it for trachoma, including reductions in prevalence or incidence of yaws, [26] genital *C*. *trachomatis* infection, [27] diarrhoea, [28] acute lower respiratory tract infection, [29] and all-cause mortality in children[30, 31]. Considered in that light, the decision of the Malawi Ministry of Health to proceed directly to delivery of a single round of MDA in this group of districts could be considered to have carried low risk and potential great benefit. Expectations of success were justifiably high–and have been borne out by this study.

The systems and methodologies that we used for training field teams, epidemiological review, fieldwork, and data processing were internationally standardized, and recognized to be of the highest quality [14]. Crucially, they were also directly comparable to the baseline surveys conducted in the same districts, in that they used the same graders, who were given refresher training in the same way as previously by the same certified trainers [17], and employed the same approaches for data acquisition and handling, from start to finish.

In between the baseline surveys [17] and the impact surveys reported here, azithromycin coverage estimates in the 8 districts, determined by dedicated surveys[15] conducted 2–4 weeks after the completion of azithromycin distribution, averaged close to the WHO-recommended minimum of 80%[11]. Interestingly—although first principles would suggest that the higher the coverage, the greater the likely impact—efforts to increase azithromycin coverage above 80% within community randomized trials have failed to show that doing so leads to greater declines (than standard-effort MDA) in the prevalence of active trachoma or ocular *C*. *trachomatis* infection[32–34]. In any event, the 69.5–83.9% coverage achieved in the 8 Malawi districts here was associated with post-MDA prevalences of TF of <5% in each of the districts’ 18 constituent EUs, and can therefore be considered, in hindsight, to have been adequate. If the low TF prevalences observed at impact survey are sustained in the absence of antibiotic pressure over the subsequent two years of surveillance [35], it may be reasonable to believe that one round of well-conducted, relatively high-coverage MDA is sufficient to eliminate active trachoma in Malawi districts that are hypoendemic at baseline.

Further north in East Africa, a cohort study in a Tanzanian community with a baseline TF prevalence in 1–9-year-olds of 36% previously suggested that one round of very high coverage azithromycin completely interrupted local transmission of ocular *C*. *trachomatis*[36, 37]. In a community-randomized trial conducted in The Gambia in an area with a baseline TF prevalence in 0–5-year-olds of 6.5%, there was no evidence that three rounds of MDA had greater impact than a single round of MDA on either the prevalence of active trachoma or ocular *C*. *trachomatis* infection [34]. One round, apparently, can sometimes be enough.

It would be remiss of us not to note that one round of antibiotics is often *not* enough [38–40]. Furthermore, combatting active trachoma should involve implementation of the A, F and E components of the “SAFE” strategy, not just the use of antibiotics [11]. Although the evidence base for the F and E components of SAFE is weaker than that for the A component, [22, 41, 42] if their combined implementation is synergistic, this could lead to gains against disease of greater amplitude and longer duration, especially after MDA is discontinued. A limitation of our study here is that we have not documented the extent to which the F and E components of SAFE were implemented in the interval between the baseline and impact surveys. In the absence of a group of control districts, we are unable to say with certainty that the observed reductions in the prevalence of TF were due to MDA, a secular trend [43–45], or something else.

From a broader public health perspective, this study provides optimism that it may indeed be possible for Malawi to eliminate trachoma as a public health problem by 2020. If this worthy goal is to be achieved, the momentum within the national programme that has been created by the leadership of government and inputs from many partners now needs to be sustained and redoubled.

## Supporting information

S1 Checklist STROBE checklist. (DOCX)
