# Supplementary material for: One round of azithromycin MDA adequate to interrupt transmission in districts with prevalence of trachomatous inflammation—follicular of 5.0-9.9%: Evidence from Malawi
Source: PLoS Negl Trop Dis. 2018 Jun 13;12(6):e0006543. doi: 10.1371/journal.pntd.0006543 (PMC6016948; doi:10.1371/journal.pntd.0006543)
Supplement: S1 Checklist — (DOCX) [file pntd.0006543.s001.docx]

STROBE Statement—report

|  | Item No | page/paragraph |
| --- | --- | --- |
| **Title and abstract** | 1 | Page 1-3 |
|  |  |  |
| Introduction | | |
| Background/rationale | 2 | Page 5-6 |
| Objectives | 3 | Page 6 paragraph 2 |
| Methods | | |
| Study design | 4 | Page 7 paragraph 2 |
| Setting | 5 | Page 7 paragraph 2 |
| Participants | 6 | Page 11, paragrapgh 1 |
| Variables | 7 | Page 11, paragraph 2 |
| Data sources/ measurement | 8* | Page 11 paragraph 2 |
| Bias | 9 | Page 12 , paragraph 1 |
| Study size | 10 | Page 8 paragraph 2 |
| Quantitative variables | 11 | Page 10 table 1 |
| Statistical methods | 12 | Page 12 paragraph 2 |
|  |  |  |
|  |  |  |
|  |  |  |
|  |  |  |
| Results | | |
| Participants | 13* | Page 12 paragrapgh 3 |
|  |  |  |
|  |  |  |
| Descriptive data | 14* | Page 12 paragraph 3 |
|  |  |  |
| Outcome data | 15* | Page 13 paragraph 1 |
| Main results | 16 | Page 13 paragraph 2 |
|  |  |  |
|  |  |  |
| Other analyses | 17 | Figure 1 |
| Discussion | | |
| Key results | 18 | Paragraph 1 page 15 |
| Limitations | 19 | Page 17 paragraph 1 |
| Interpretation | 20 | Page 17 paragraph 2 |
| Generalisability | 21 | Page 17 paragraph 2 |
| Other information | | |
| Funding | 22 | Page 17 paragraph 3 |
